# Reframing youth suicide as a public health crisis: Where does accountability lie?

**DOI:** 10.1371/journal.pgph.0007368

**Published:** 2026-10-14

**Authors:** Ananya Mosur Sridhar, Parth Sharma

**Affiliations:** 1 Department of Clinical, Education and Health Psychology, University College London (UCL), London, United Kingdom; 2 Initiative for Health Equity Advocacy and Research (IHEAR), Sangath, Bhopal, Madhya Pradesh, India; PLOS: Public Library of Science, UNITED STATES OF AMERICA

## Youth suicides in India: A public health crisis

Suicide is one of the leading causes of death among young people in India (a Low- and Middle-Income Country), with 71% female, and 58% male deaths by suicide occurring between ages 15–39 [1]. This growing burden of youth suicides in India fundamentally challenges a predominant assumption within the discourse of suicide prevention, one where ‘individual pathology’ is misconstrued as the principal target for intervention. While mental ill health remains an important risk factor, it explains only part of a complex phenomenon of suicide, which is shaped by several sociopolitical, economic and gendered indicators [2]. This framing of a youth mental health ‘epidemic’ that places the onus of suicide on a young person has inadvertently siloed the discourse to clinical interventions. With the recent student deaths surrounding the NEET examinations in India and the ongoing student protests, schools are re-entering the suicide prevention discourse along with several other necessary interest-holders although they run the risk of being seen in isolation as well. Reframing youth suicides in India through a public health lens becomes a necessary task towards interrogating and further dispersing collective accountability. However, none of this is possible without foregrounding young people with multiple marginalized identities as the key knowledge producers.

Today, the language around suicide prevention in global public health is often presented as a universal, value-neutral phenomenon where interventions are designed for holistic well-being. Yet the foundations of this movement borrow from colonial histories, where self-inflicted death as an act of liberation among enslaved and colonized peoples was routinely interpreted through biopolitical frameworks centred on preservation of enslaved labour, state ownership over bodies, and pathologization of anti-colonial resistance [3]. While that framing has substantially evolved, the insistence on bio-medical models to understand suicide continues to obscure present structural inequities of rampant militarization, caste violence, Indigenous land erasure, ableism and anti-trans discrimination that young people in India continue to face.

India accounts for nearly 18% of the global population, but approximately for 28% of global suicides. The prevalence of suicide in India is higher than the global average and while the Mental Health Care Act 2017 and the National Suicide Prevention Strategy (NSPS) 2022 emerged as public health priorities, their implementation remained weak due to a lack of coordinated intersectoral collaboration and in many cases political will at both the national and regional level [1, 4]. This is further evidenced by the surge in youth suicides in India, especially those occurring around examinations like the National Eligibility-Entrance Test (NEET) [5, 6].

### Lack of intersectionality in youth mental health

De-politicized narratives around youth mental health flatten identity to one axis - age - and homogenize youth as a singular category. Problematizing this framing is necessary to understand the heterogeneity of experience within India, and moreover, to understand the impact of institutional violence on specific communities. For several years, young people from oppressed communities (Dalit, Bahujan, Adivasi, Queer, Trans, Disabled, Muslim, and geographically Othered communities) have advocated for reform within the Indian education system, as a matter of human rights. S. Anitha was a 17-year-old Dalit student from a government school in a low-resource setting in Tamil Nadu who died by suicide in 2017 after resisting NEET (an examination that can be argued to be rooted in meritocracy over equity) in the Supreme Court, only to be refused justice. Rohith Vemula, a 26-year-old Dalit PhD student, died by suicide in 2016 after resisting caste injustice on campus, a year prior to S. Anitha [7]. Their deaths are two of several such examples of institutional violence that continue to remain detached from the larger systemic accountability around youth suicides.

In the process of reframing youth suicide as a public health crisis, and foregrounding lived experience it becomes imperative that we work through the lens of intersectionality.

### Placing systemic accountability: Examining the role of state

Youth suicides and in extension mental health in India is often met with ‘a firefighting response’; where care infrastructures emerge from grassroots, and Civil Society Organizations (CSOs) overextend their role as defacto intermediaries offering crisis interventions, psychosocial support and aid [8]. For some communities (such as LGBTQIA+) CSOs fill a gap that the state refuses to acknowledge, placing the burden of care on already stretched organizations, especially those heavily reliant on foreign funding [9].

Informal care infrastructures are not new to India. Across Public Health Emergencies like COVID-19 pandemic, several collectives emerged as short term, accessible pathways to mobilise support, ranging from mutual aid campaigns, to pro-bono psychosocial and legal aid [10]. Today these services are re-emerging as youth suicides garner more visibility; however, they are also creating un-intended consequences of burnout within CSOs that were never created to meet the demands of today’s youth. While acknowledging this overextended role of CSOs is important, it is equally necessary to ask why the state is rarely held accountable.

Take National Education Policy (NEP) 2020 Positioned as a paradigm shift in Indian education, it fell flat, with very minimal foresight into implementation. On paper, it aimed at strengthening institutional capacity to respond effectively during youth mental health crises through counsellors and mental health interventions. However, its roll out did not consider the lack of infrastructure in schools and higher education institutions across low-resource settings [11]. Six years since its launch, entire communities continue to be excluded from conversations around access to education and psychosocial support- especially those that are queer, trans, disabled, currently unhoused, incarcerated, living under active armed conflict and increased militarization, forcibly displaced or under immediate climate crises.

Student suicides and an unjust education system can then not be seen as mutually exclusive.

### Youth suicide prevention in the post-decriminalization era

Only recently has India removed the provisions criminalizing attempted suicide through Bharatiya Nyaya Sanhita (BNS) 2023 [12]. The law explicitly endorses decriminalization; however, it does not automatically lead to the destigmatization of suicide at the grassroots level. Today, individualistic conceptualization of resilience, where suicide prevention strategies often created without consultation with the youth use a reductionist gaze placing the young person to have a deficit in need of correction [13]. Reframing youth suicides as a public health crisis requires a systems approach where young people with intersectional lived experience are at the forefront of change, interventions are community led, decentralized, and follow an intersectoral collaboration across media, law, social work, health services and public policy [14, 15].

A systems approach without formal methods of accountability runs the risk of abstraction and has potential to remain tokenistic The central question around youth suicide needs to shift from “*what went wrong in a young person’s life?”* to “*what systems were complicit in their death?*”. States must be held accountable for implementation gaps of rights-based frameworks; caste violence in education institutions, anti-trans discrimination, ableist policies, and the routine curbing of dissent. At the same time, public health researchers and CSOs must ensure that intersectionality of lived experience remains central to intersectoral collaboration, and that dispersion of accountability in youth suicide prevention is defined and evaluated. Youth mental health is inherently political, and every time a young person dies by suicide due to oppressive and unjust systems, we are reminded that their death was avoidable. We must centre accountability as the organizing principle of youth suicide prevention.
